# Increasing Pediatric Infectious Diseases Consultation Rates for *Staphylococcus aureus* Bacteremia

**DOI:** 10.1097/pq9.0000000000000560

**Published:** 2022-06-14

**Authors:** Oren Gordon, Nadine Peart Akindele, Christina Schumacher, Ann Hanlon, Patricia J. Simner, Karen C. Carroll, Anna C. Sick-Samuels

**Affiliations:** From the * Division of Infectious Diseases, Department of Pediatrics, Johns Hopkins University School of Medicine, Baltimore, Md.; † Center for Child and Community Health Research, Department of Pediatrics, Johns Hopkins School of Medicine, Baltimore, Md.; ‡ Division of Medical Microbiology, Department of Pathology, Johns Hopkins University School of Medicine, Baltimore, Md.

## Abstract

**Introduction:**

*Staphylococcus aureus* bacteremia (SAB) in children is associated with significant mortality and morbidity, including recurrent bacteremia. Infectious disease consultation (IDC) improves SAB outcomes in adult patients. However, increasing IDC and impact for pediatric patients with SAB is not well described.

**Methods:**

This quality improvement project aimed to increase IDC for SAB events at a quaternary pediatric medical center. First, we evaluated the local practices regarding pediatric SAB and engaged stakeholders (July 2018–August 2020). We added an advisory comment supporting IDC for SAB to all blood culture results in September 2020. Using statistical process control charts, we monitored the number of SAB events with IDC before a SAB event without IDC. Finally, we evaluated SAB recurrences before and after initiating the advisory comment.

**Results:**

In the baseline period, 30 of 49 (61%) SAB events received an IDC with a mean of 1.4 SAB events with IDC before a SAB event without IDC. Postintervention, 22 of 23 (96%) SAB events received IDC with a mean of 14 events with IDC before 1 event without IDC. The SAB recurrence rate was 8%, with 6 events in 4 children; none of the index cases resulting in recurrence received an IDC (*P =* 0.0002), and all occurred before any intervention.

**Conclusions:**

An electronic advisory comment supporting IDC for SAB significantly increased the rate of pediatric IDC with no further SAB recurrence episodes following intervention. This low-resource intervention may be considered in other pediatric centers to optimize SAB management.

## INTRODUCTION

*Staphylococcus aureus* bacteremia (SAB) is associated with significant mortality and morbidity in children, including disseminated disease, secondary foci of infection, and recurrent infection.^1–5^ Recurrence of SAB, defined as relapse or re-infection, may occur even months after the initial episode despite treatment with antibiotics. Factors linked to recurrence of SAB in adult patients include persistence of a deep-seated infection, immunosuppression, previous liver or renal disease, and duration of parenteral therapy for catheter-related bacteremia less than 10 days.^6–8^

Adherence to evidence-based measures to treat SAB can improve outcomes, including repeating blood cultures to document clearance, early source control, echocardiography, and appropriate selection and duration of antibiotic therapy. Infectious disease consultation (IDC) improved adherence to guidelines in managing adult patients with bacteremia.^9,10^ IDC improved patient outcomes when treating SAB.^11–19^ Early IDC within the first 48–72 hours of admission lowered 30-day mortality and the rate of readmission and hospital and intensive care unit length of stay among adult patients.^20,21^

Few studies have evaluated the impact of IDC on bacteremia among children. However, initial reports suggest a similar benefit to pediatric patients.^22^ Pediatric IDC was associated with a higher rate of appropriate therapy and may have decreased mortality due to enterococcal bacteremia in children.^23^ In another study, children with SAB who received IDC were more likely to have an identified removable focus of infection, get an echocardiogram, and receive a longer course of intravenous antibiotics.^24^ However, there were no differences in outcomes regarding recurrence and mortality, presumably due to the small sample size. Furthermore, few studies evaluated approaches to improve IDC rates in children. Our division was aware of the growing evidence that IDC may improve SAB management, and preliminary data from our hospital suggested many patients with SAB had not received IDC. Additionally, several children with SAB recurrences treated in our center had not received IDC during the index SAB event, raising the question of whether there were opportunities to improve management of the initial infection. This project aimed to increase IDC for patients with any SAB in our center and to assess whether this would impact SAB recurrence in children.

## METHODS

### Context

This quality improvement project was initiated by infectious disease fellows at the Johns Hopkins Children Center (JHCC), a 205-bed pediatric quaternary referral center in Baltimore, Maryland, with 40 and 45 pediatric and neonatal intensive care unit (PICU and NICU) beds, respectively. The Division of Pediatric Infectious Diseases is part of the Department of Pediatrics at JHCC and consults by requests initiated by primary team clinicians. Primary teams include general pediatrics, PICU, NICU, pediatric emergency medicine, general pediatric surgery, pediatric orthopedics, and pediatric subspecialties. Annually, the pediatric ID division completes about 500 new consultations. The study included all patients with *S. aureus* identified in at least 1 blood culture obtained at the JHCC from July 2018 through July 2021. The presentation follows the SQUIRE 2.0 Guidelines.^25^

### Quality Improvement Cycles

**Fig. 1.**
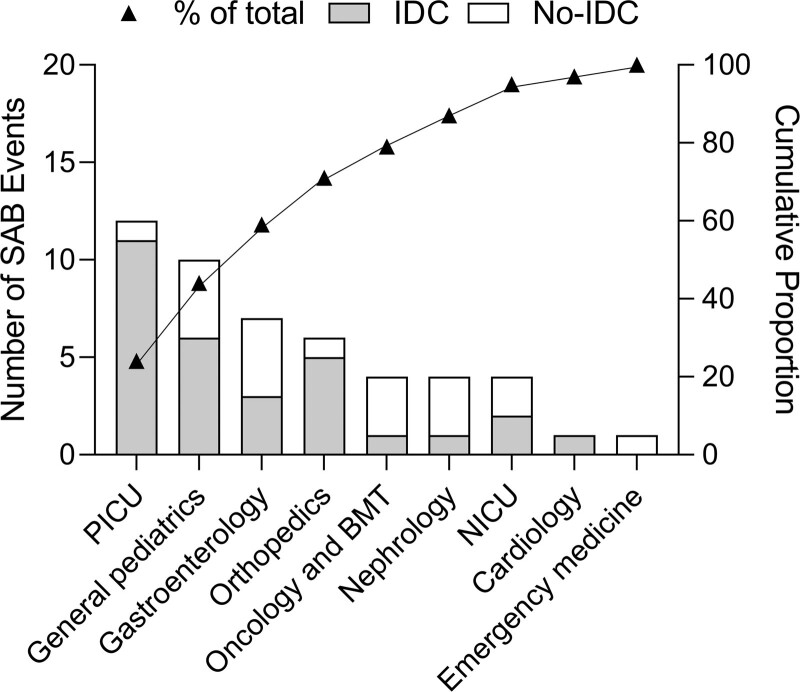
Pareto chart showing the utilization of infectious diseases consultation by primary clinical teams before intervention. BMT, bone marrow transplantation.

**Fig. 2.**
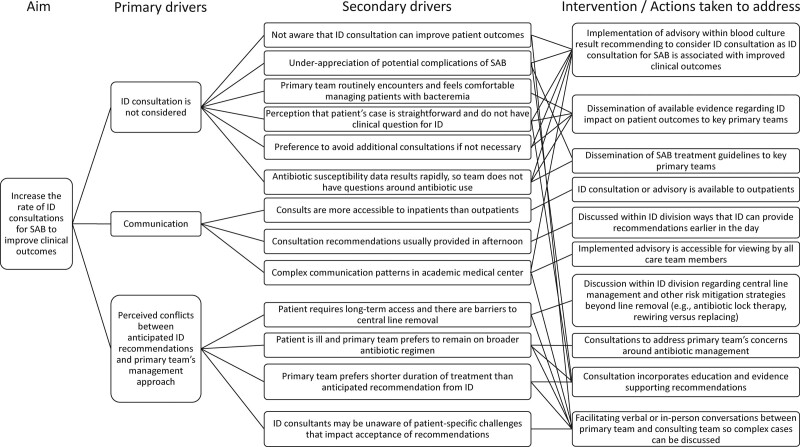
Contributing drivers and potential solutions regarding consultation with pediatric infectious diseases for *Staphylococcus aureus* bacteremia. ID, infectious diseases.

We initially aimed to increase the number of SAB events that received IDC by 20% within 1 year to optimize SAB management and reduce complications of SAB recurrence. As a result, the QI team completed 3 plan-do-study-act cycles as follows:

*1. QI Team formation and formative data collection.* Our team included two pediatric ID fellows and 2 pediatric ID attendings. Our first objective was to evaluate the rate of IDC for SAB events in our institution and to understand the characteristics of SAB events that received IDC. Before the study intervention, we evaluated the IDC utilization rate for SAB events by different primary clinical teams from July 2018 to April 2020. The intensive care and surgical teams had higher ID utilization rates (88% and 83%) than the medical services (59%). We identified 5 primary teams that may benefit from targeted intervention based on lower utilization rates (gastroenterology, oncology, bone marrow transplant, and general pediatrics) or higher rates of SAB occurrence (PICU) (Fig. 1).
From April to September 2020, our team initiated open communication with physician fellows, attendings, and division heads of the 5 identified groups to increase awareness for the project. Twelve fellows were surveyed regarding specific hurdles in obtaining IDC for SAB. Eight fellows completed the survey (67% response rate). We present a summary of the identified drivers and solutions in Figure 2. Three primary drivers were identified. (1) Primary teams did not consider consulting ID because of low awareness of the potential complexity of SAB and the benefit of IDC. (2) Communication gaps existed between primary and ID teams related to different working patterns (morning versus afternoon rounds, outpatients versus inpatients, etc.) (3) The potential conflict anticipated between ID recommendations and primary team’s management approach (eg, expecting ID would recommend removing central line when the patient is central line-dependent and with difficult vascular access). The fellows and faculty of our ID division discussed the feedback provided and the potential solutions.
*2. Education of primary teams.* Recognizing a major driver for not getting IDC was unawareness of the benefit of IDC, we provided education to all primary teams at JHCC from April 2020 to September 2020. This education included presentations at resident physician conferences, in-person and emailed education provided to fellows and attendings, and presentation at a department of pediatrics quality improvement meeting. All education formats emphasized that IDC has been associated with better outcomes in SAB events based on published data in adults. In addition, we monitored the IDC rate for SAB events for the subsequent quarter. There was no change in the consultation rate following cycles 1 and 2. Therefore, we considered a more sustainable intervention that would promptly reach the most relevant front-line clinicians.
*3. Implementing advisory comment.* Collaborating with the microbiology laboratory, on September 1, 2020, we implemented an advisory comment to the Laboratory Information System (SoftLab; SCC Soft Computer Consultants, Clearwater, Fla.) report that crossed into the electronic medical record (EMR)(EPIC; Epic Systems, Verona, Wis.) report of any positive blood culture with *S. aureus* obtained in the pediatric emergency or inpatient wards. The comment states, “Pediatric Infectious Disease (ID) consultation for *S. aureus* bacteremia has been shown to improve patient outcomes. Consider discussing the results of this blood culture with the pediatric ID service.” We continued to monitor IDC for SAB quarterly, from April 1, 2020, through July 30, 2021. We shared the collected data with the pediatric ID division and solicited feedback on the changes. No further process changes were made, given the robust response to the advisory comment.

### Measures

We defined our primary process measure as the number of SAB events that received an IDC before a SAB event that *did not* receive an IDC. A sign of improvement would be that more SAB events with IDC would occur before a SAB event without IDC. This approach is similar to following the number of days between surgical site infections after improving surgical wound care practices. Because the rate of SAB is relatively infrequent, we followed the cumulative number of SAB events rather than days elapsed.

We selected SAB recurrence as the primary clinical outcome because it is a complication of SAB that may be preventable with optimized management.^26^ SAB recurrence was defined as a positive blood culture taken over 28 days and within 6 months following the index episode. The isolates had to have a similar antibiotic susceptibility pattern.

Additional process outcomes included if the administered antibiotic was appropriate at the following time points: when the initial blood culture was taken (time 0) and after 12, 24, 48, 72, 96, and 120 hours. We defined the appropriate *empiric* antibiotic treatment (before the availability of full *in vitro* antibiotic susceptibilities) based on the known Johns Hopkins Children Center antibiogram for *S. aureus* blood isolates: vancomycin, daptomycin, and linezolid for methicillin-resistant *S. aureus* (MRSA) and either oxacillin, cefazolin, meropenem, cefepime, piperacillin/tazobactam or ampicillin/sulbactam for methicillin-susceptible *S. aureus* (MSSA). We defined appropriate *definitive* antibiotic treatment based on *in vitro* antibiotic susceptibilities of the culture result. We considered the initial episode as all the blood cultures with *S. aureus* occurring during the initial antibiotic course or within 14 days of the first positive culture.

We identified all SAB infections by EMR query of culture results quarterly. In addition, we performed a chart review of all SAB cases to obtain the following data: age, sex, primary clinical service, prior comorbidities, genus and species of organism recovered from blood, antibiotic susceptibility profiles, duration of bacteremia (from first positive to first negative blood culture), ICU admission, infectious foci/complication, duration of intravenous and total antibiotics, duration of hospital stay after first positive blood culture, all-cause mortality, whether IDC was obtained, time to IDC from when blood culture was taken, whether IDC led to any recommendations regarding the removal of infectious foci, repeat blood cultures, change of antibiotics and duration of antibiotics.

### Laboratory Methods

The Johns Hopkins Hospital Medical Microbiology Laboratory processed the blood cultures and completed bacterial identification and antibiotic susceptibility testing of isolated organisms. The laboratory notified primary clinicians of Gram stain results throughout the study period as soon as they were available. The laboratory uses Verigene Gram-Positive Blood Culture multiplex nucleic acid test (Luminex, Austin, Tex.) to identify Gram-positive organisms to the genus and/or species level and associated antimicrobial resistance markers, including identification of *S. aureus* and *mecA*. The laboratory performed the Gram-Positive Blood Culture assay only on the first blood culture growing Gram-positive cocci within 7 days and reported results within 4 hours of the Gram stain result, as previously described.^27^ The laboratory reported *S. aureus* blood cultures as MRSA if the *mecA* gene was detected and MSSA if not. All positive blood cultures were subcultured to solid media to identify the pathogen using matrix-assisted laser-desorption ionization time-of-flight mass spectrometry (Bruker Daltonics Inc, Billerica, Mass.) as necessary and antibiotic susceptibility testing using the BD Phoenix Automated System (BD Diagnostics, Sparks, Md.). The laboratory reported Gram-Positive Blood Culture, identification, and/or AST results in the EMR as results became available.

### Data analysis

We used a statistical process control chart, specifically a G-chart, to plot the number of SAB events with IDC before a SAB event without a consultation against the cumulative total number of SAB events. We also monitored the proportion of SAB with IDC per quarter using a P-chart. The baseline centerlines of each chart were defined using data before any interventions began (July 2018–June 2020). We considered rules for special cause variation,^28^ and shifted the centerline when evidence of a clear and sustained shift in practice. We compared SAB recurrence rates pre- and postintervention using the 2-tailed Fisher exact text. Demographic and clinical characteristics were compared between SAB events with or without IDC using a 2-tailed Fisher exact text for categorical variables, a 2-tailed Mann-Whitney test for continuous variables, and 2-way analysis of variance for the time from blood culture to adequate antibiotic coverage. *P* ≤ 0.05 was considered significant for all analyses. Analysis was performed using GraphPad Prism 9 (GraphPad Software) or Stata version 13 (StataCorp, College Station, Tex.).

### Ethical considerations

The Johns Hopkins Hospital Institutional Review Board acknowledged the study as quality improvement with a waiver of informed consent. All data were collected and stored on a monitored, firewall-protected server and accessible only to the necessary study team members.

## RESULTS

### ID Consultation for SAB

**Fig. 3.**
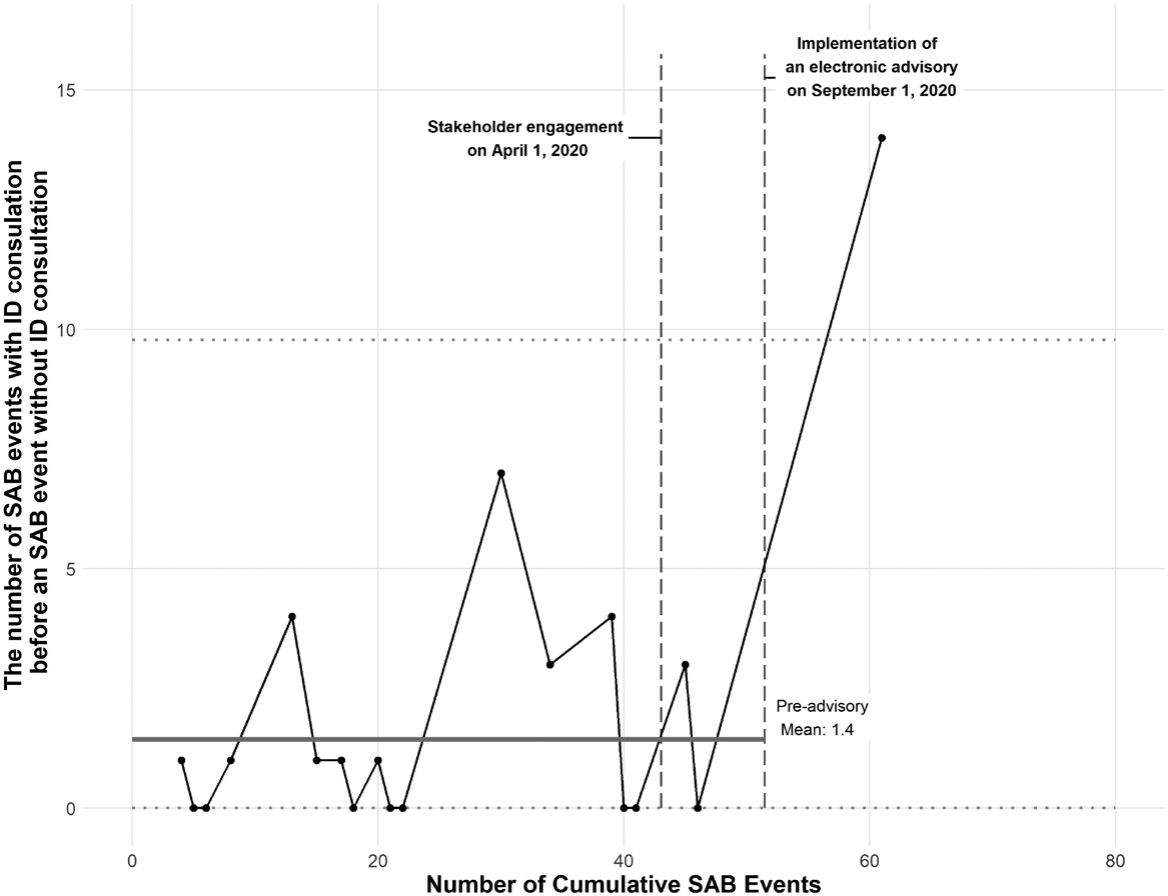
G-chart of the number of *S. aureus* bacteremia events before 1 without a consultation versus the cumulative number of *S. aureus* bacteremia events. Solid gray line represents the preintervention mean number of SAB events before 1 with no consultation. Dotted horizontal lines are the upper and lower confidence intervals (3 sigma). Vertical dashed lines mark the study timeline with stakeholder engagement and education beginning April 2020 and implementation of an electronic advisory beginning September 2020.

**Fig. 4.**
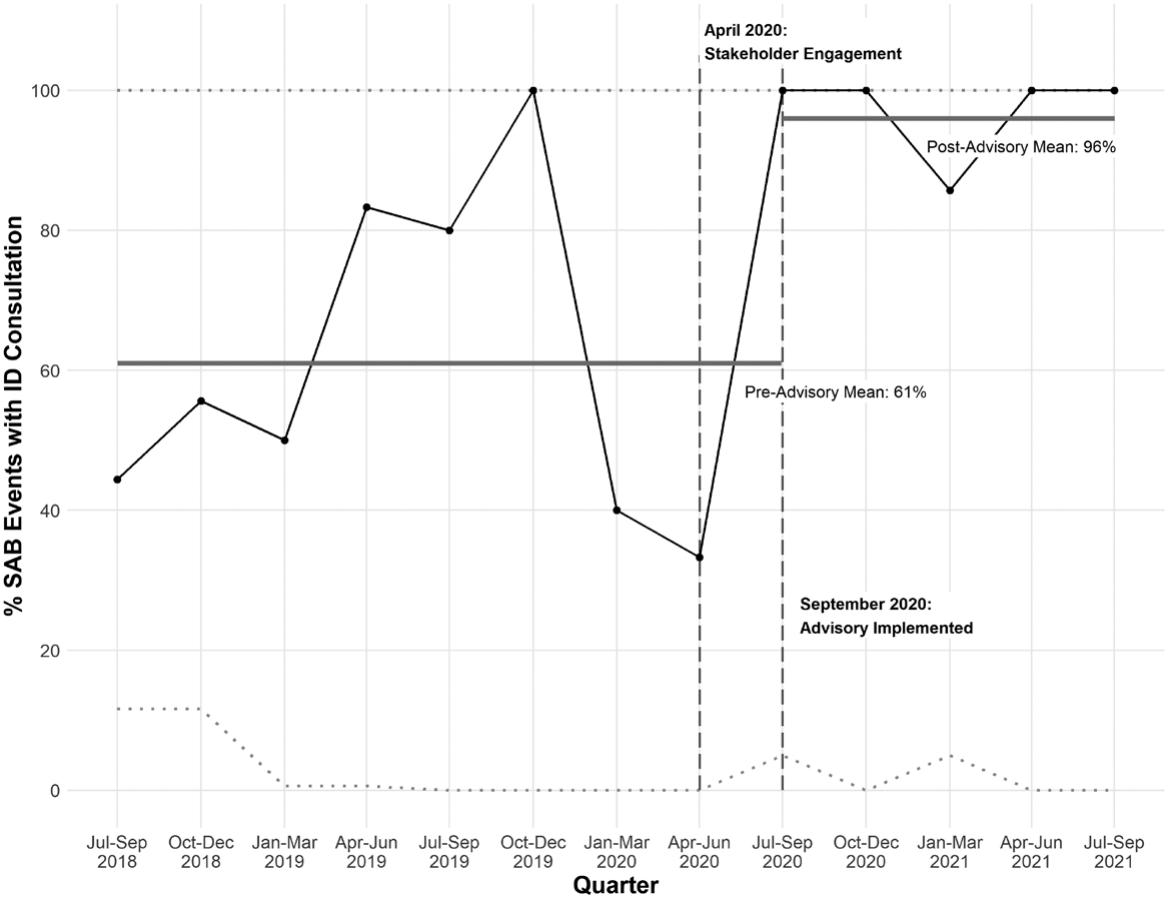
Proportion of pediatric infectious diseases consultation for *Staphylococcus aureus* bacteremia events, before and after implementation of an electronic advisory. Solid black line represents the proportion of events receiving ID consultation by quarter (the last data point is for July 2021 only). Solid gray lines represent the mean consultation rate pre- and postintervention. Dotted horizontal lines are the upper and lower confidence intervals (3 sigma). Vertical dashed lines mark the study timeline with stakeholder engagement on April 2020 and implementation of an electronic advisory on September 1^st^ 2020.

We observed seventy-two SAB episodes in 68 children within the study period. The average monthly SAB event rate was similar across the study period (2.0 per month from July 2018 through August 2020; 2.1 per month from September 2020 to July 2021). The median age of patients was 6.7 years (interquartile range [IQR] 0.9–13.3), with 48% being females. Of the 72 SAB events, 52 (72%) received an IDC, and 20 (28%) did not. The mean number of SAB events that received IDC before a SAB event that did not receive a consultation increased significantly from 1.4 events before the intervention to 14 events after the intervention, which was greater than 3 standard deviations above the baseline mean (Fig. 3). Similarly, there was a significant increase in the proportion of SAB events with IDC following the implementation of the advisory comment in September 2020: preintervention, 30 of 49 SAB events received IDC (61%) compared with 22 of 23 postintervention (96%) (Fig. 4). In the 1 SAB case that did not receive IDC postintervention, the primary team verbally discussed the case with the ID team without formal consultation.

Regarding the additional process measures, adequate empiric antibiotic coverage during blood cultures were drawn was given to 70% and 79% of children with or without IDC, respectively (*P =* 0.56). After obtaining a blood culture with or without IDC, the proportion of adequate antibiotic coverage reached 96% versus 95% by 48 hours and 100% versus 95% by 96 hours, respectively (*P =* 0.61).

### SAB Recurrence

The overall SAB recurrence rate was 8%, with 6 events in 4 children. Importantly, none of the index cases resulting in recurrence received an IDC (*P =* 0.0002). All index SAB events resulting in recurrence occurred before intervention. All the index events were central-line-associated bloodstream infections in central line-dependent children receiving total parenteral nutrition or hemodialysis (Table 1). Half of the recurrences were due to methicillin-resistant *S. aureus* (MRSA). During the index SAB event, the initial presentation was not severe, and none of the children was admitted to the PICU. The central line was not removed in 4 of 6 index cases. SAB recurrence was complicated in 1 child by significant secondary seeding of infection, including pulmonary septic emboli and osteomyelitis (patient 3 in Table 1).

**Table 1.** Characteristics of *Staphylococcus aureus* bacteremia recurrence within 6 months of index event (July 2018–July 2021)^*^

	Patient 1			Patient 2			Patient 3			Patient 4		
History	6-year-old, ESRD on hemodialysis			17-year-old, Chronic intestinal pseudo-obstruction, TPN-dependent			4-year-old, Short bowl syndrome, TPN-dependent			9-year-old, Short bowl syndrome, TPN-dependent		
Index case and subsequent recurrence(s)	1^st^ SAB	2^nd^ SAB	3^rd^ SAB	1^st^ SAB	2^nd^ SAB	3^rd^ SAB	1^st^ SAB	2^nd^ SAB	3^rd^ SAB	1^st^ SAB	2^nd^ SAB	3^rd^ SAB
MRSA	N	N	—	Y^†^	Y^†^	Y^†^	N	N	N	Y	Y	—
Primary focus of infection	Line	Line	—	Line	Line	Line	Line	Line	Line	Line	Line	—
Days since previous SAB (d)	—	61	—	—	161	122	—	64	65	—	111	—
ID consulted	N	Y	—	N	N	Y	N	N	Y	N	N	—
Time to appropriate antibiotics (h)	12	0	—	0	0	0	0	0	0	0	24	—
Time to negative blood culture (d)	3	3	—	3	1	1	2	Not taken	4	6	2	—
Duration of antibiotics (days)^‡^	14	16	—	17	14	14	8	10	44	17	7	—
Infected line removed^§^	Y	Y	—	Y	N	N	N	N	Y	N	N	—
Lock therapy	N	N	—	N	N	Y	Y	N	N	Y	Y	—
Secondary foci of infection	N	N	—	N	N	N	N	N	Y^¶^	N	N	—

*All index events took place before study intervention.

†Known colonization with MRSA. Decolonization protocol was performed following ID consultation.

‡All antibiotic treatment was given intravenously.

§For all cases where infected line was removed, it was immediately followed by insertion of a new central line

¶Hip osteomyelitis and septic pulmonary emboli.

ESRD, end stage renal disease; MRSA, methicillin-resistant *Staphylococcus* aureus; SAB, *S. aureus* bacteremia; TPN, total parenteral nutrition.

### Comparing SAB with or without ID consultation

To better understand the clinical impact of IDC, we compared SAB events with and without IDC (Table 2). A primary focus of infection was identified in most patients (76%), and the most prevalent primary focus was a line infection (51% of all cases). Most line infection cases received IDC (65%). There were overall 10 cases of endovascular infection (14%), including 6 cases of endocarditis (8%), and all of these received IDC (Table 2). MRSA was the causative pathogen in 14 (19%) cases, of which 9 (64%) received IDC.

**Table 2.** *Staphylococcus aureus* Bacteremia Events with and without ID Consultation (July 2018–July 2021)

Characteristics	Total^*^N (%)	ID Consultation N (%)	No ID Consultation N (%)	*P*
Number	72 (100%)	52 (72%)	20 (28%)	
Age, median years (IQR)	6.7 (0.9–13.3)	7.1 (0.8–13.4)	6.2 (1.3–11.9)	0.85
Female	35 (49%)	23 (44%)	12 (60%)	0.30
Male	37 (51%)	29 (56%)	8 (40%)	0.30
Primary team				0.07
Medical	29 (40%)	17 (33%)	12 (60%)	0.06
Surgical	12 (17%)	10 (19%)	2 (10%)	0.49
ICU	24 (33%)	21 (40%)	3 (15%)	0.05
Oncology/bone marrow transplant	7 (10%)	4 (8%)	3 (15%)	0.39
Any prior diagnoses	55 (76%)	37 (71%)	18 (90%)	0.12
Gastrointestinal disease	11 (15%)	6 (12%)	5 (25%)	0.27
Malignancy	11 (15%)	7 (13%)	4 (20%)	0.49
Genetic syndromes	8 (12%)	8 (17%)	0 (0%)	0.10
Cardiac disease	8 (12%)	8 (17%)	0 (0%)	0.10
Pulmonary disease	8 (12%)	4 (9%)	4 (20%)	0.21
Renal disease	4 (6%)	2 (4%)	2 (10%)	0.31
Other	16 (22%)	11 (21%)	5 (25%)	0.76
MRSA	14 (19%)	9 (17%)	5 (25%)	0.76
Hospital acquired^†^	24 (33%)	19 (37%)	5 (25%)	0.41
Community acquired^†^	48 (67%)	33 (63%)	15 (75%)	0.41
Fever	61 (85%)	43 (83%)	18 (90%)	0.71
WBC, median cell count × 10^3^/mm^3^ (IQR)	11.2 (5.9–17.7)	11.4 (6.2–19.0)	9.4 (3.3–13.0)	0.03
CRP, median mg/dL (IQR)	7.0 (3.1–17.5)	9.7 (4.9–21.8)	3.4 (1.2–5.1)	0.002
Any foci of infection^‡^	55 (76%)	40 (77%)	15 (75%)	0.99
Line infection	37 (51%)	24 (46%)	13 (65%)	0.19
Osteoarticular infection	24 (33%)	18 (35%)	4 (20%)	0.27
Endovascular infection^§^	10 (14%)	10 (19%)	0 (0%)	0.05
Respiratory tract infection	3 (4%)	2 (4%)	1 (5%)	0.99
Time to positive blood culture, median hours (IQR)	17 (14.0–23.0)	18 (14.0–23.0)	17 (13.3–23.3)	0.87
Time to ID consultation, median days (IQR)	–	1.4 (0.1–2.0)	NA	NA
Time to first negative culture, median days (IQR)	1.9 (1.0–3.0)	2.0 (1.5–3.9)	1.0 (1.0–3.0)	0.02
Length of stay after first positive culture, median days (IQR)	11.6 (4.6–23.6)	13.3 (5.7–24.4)	5.3 (1.7–14.9)	0.02
Duration of IV antibiotics, median days (IQR)	14 (7–27.5)	14.5 (7–32)	10 (6–16)	0.04
Primary line infection	14 (13.3–16)	14 (13.5–15)	14 (10–17)	0.63
Osteoarticular infection	6 (3–13.7)	7 (4–14)	2 (1.5–7.5)	0.05
Endovascular infection	44.5 (40.3–49.5)	44.5 (40.3–49.5)	NA	NA
Duration of total antibiotics, median days (IQR)	28 (14–42)	30 (15–44)	14 (9–17)	0.0002
Primary line infection	14.5 (14–16)	15 (14–15.5)	14 (10–17)	0.09
Osteoarticular infection	34.5 (28–44)	38 (28–45)	28 (8–40)	0.10
Endovascular infection	44.5 (40.3–49.5)	44.5 (40.3–49.5)	NA	NA
Relapse with *S. aureus* bacteremia within 6 mo	6 (8%)	0 (0%)	6 (30%)	0.0002
Death	4 (6%)	3 (6%)	1 (5%)	0.99

*Total 68 children with 72 events.

†An infection was considered community acquired if the positive culture was drawn on or after the 3^rd^ calendar day of admission where day of admission is calendar day 1

‡One event may have more than one focus of infection.

§Endovascular infections did not include central-line associated infections with only bacteremia

MRSA, methicillin-resistant *Staphylococcus* aureus; NA, not applicable.

Children who received IDC presented with higher inflammatory markers and had a longer duration of bacteremia (2 days versus 1 day, *P =* 0.02). They had a longer length of admission following bacteremia (13 days versus 5 days; *P =* 0.02). The total antibiotic treatment duration was longer with IDC (30 versus 14 days [*P =* 0.0002]). However, ID was typically consulted for the diagnoses that require treatment courses beyond 14 days (eg, endovascular and osteoarticular infections) (Table 2). The duration of intravenous antibiotics was significantly longer when ID was consulted (median duration: 14.5 versus 10 days [*P =* 0.04]).

When considering the IDC recommendations, among 52 total consultations, 51 (98%) included recommendations for the duration of antibiotics, 49 (94%) requested repeat blood cultures, 46 (88%) recommended a change in antibiotics, 25 (48%) suggested removing an infectious focus, and 26 (50%) recommended additional imaging, including an echocardiogram.

## DISCUSSION

Implementing an advisory comment into the blood culture results notifying first-line clinicians that IDC may improve outcomes for patients with SAB was associated with a dramatic shift in practice such that 96% of children with SAB received IDC. Before the advisory was implemented, we solicited feedback to understand IDC drivers and provided education regarding the benefits of IDC to primary team clinicians. These formative steps and stakeholder engagement may have helped bring attention and acceptance of the advisory supporting IDC for SAB, as was recently described.^29^

The primary clinical outcome, recurrence within the first 6 months, occurred in 8% of SAB events, none of the index events had received IDC, and all occurred before intervention. Recurrence occurred only among children dependent on central lines for total parenteral nutrition or dialysis. In these children, vascular access may be limited, and removal of central lines with disruption of care and feasibility of replacement must be balanced against the risk of recurrent infection, which may also be life-threatening. The benefit of IDC may relate to recommendations around antibiotic duration, addressing source control, or seeking additional imaging to identify secondary infectious complications. Indeed, in index cases leading to recurrence that did not receive IDC, the duration of the antibiotic was less than 14 days in 33%, and the infected line was not removed in 67%. When engaging with primary teams, some indicated they may not have consulted ID if they anticipated infeasible recommendations (ie, line removal in line-dependent child). Importantly, the consulting teams must discuss intervention risks, benefits, and options that may help reduce the risk of secondary infectious complications. In our project, we did not evaluate the impact of IDC on time to line removal or the use of alternative mitigation methods such as guidewire replacement.^30^

For adult SAB events, the recurrence rate following antibiotic therapy is 5%–12%^6^ and is reduced by IDC.^26^ A recent prospective cross-sectional study by Campbell et al found that 4% of children with SAB had a relapse within 3 months.^31^ In that study, IDC was protective against mortality related to SAB (adjusted odds ratio 0.07 with 95% confidence interval [95%CI] 0.004–0.9).^31^ In another recent study, Duguid et al retrospectively compared the outcome of pediatric SAB cases with and without IDC. They found that IDC was independently associated with cure, defined as the absence of death or relapse at 30 days (odds ratio 31.5. 95%CI 1.2–845.0; *P =* 0.04).^32^ Our findings are consistent with these studies and support that IDC is protective against SAB recurrence.

This study has several limitations. First, this is a single-center evaluation in a quaternary pediatric center. Results may reflect the unique patient population, including highly complex patients, and may be influenced by the small sample size. However, SAB can occur across pediatric populations, and our approach should be relevant to other pediatric hospitals. Second, we assumed recurrence of SAB represents relapse with the same isolate. However, it is plausible that re-infection, or new infection, could occur and that any interventions targeting the index infection would not impact their occurrence. We defined recurrence as an episode of SAB with a similar antibiotic susceptibility pattern but cannot definitively assert whether the bacterial isolates were identical or independent infections. Still, in a large multi-center prospective study, 79% of recurrences within 6 months following SAB were classified as relapses, and only 21% as re-infection, based on pulsed-field gel electrophoresis pattern.^6^ Third, we may have underestimated the recurrence rate if patients sought care elsewhere, though most complex patients return to their medical homes. Lastly, we did not obtain formal feedback from primary teams nor evaluate balancing measures such as cost to patients for the additional consultation or patient satisfaction. We solicited feedback from the ID division faculty and fellows, who supported the continuation of the advisory comment and did not give negative feedback regarding increased consultations for SAB, which constituted a relatively minor proportion of 2% of annual consultations.

In conclusion, implementing an advisory comment to consider IDC for SAB embedded in the blood culture report significantly increased the rate of pediatric IDC for SAB, and there were no additional SAB recurrence events following the intervention. This intervention is low-resource and may lead to improved clinical outcomes. Future studies are needed to elucidate the optimal management of vascular access following SAB in central-line-dependent children and assess if there could be a benefit of a similar EMR-based intervention in other settings such as outpatient-based laboratories or centers without immediate access to ID specialists.

## DISCLOSURE

The authors have no financial interest to declare in relation to the content of this article.

## ACKNOWLEDGMENT

We wish to dedicate this article in loving memory of Dr. Kwang Sik Kim, a leader in his field of Pediatric Infectious Diseases, who was dedicated to the training of many generations of ID fellows.
